# The foundations of influencing policy and practice: How risk science discourse shaped government action during COVID‐19

**DOI:** 10.1111/risa.14213

**Published:** 2023-09-02

**Authors:** Duncan Shaw, Judy Scully

**Affiliations:** ^1^ Alliance Manchester Business School The University of Manchester Manchester UK; ^2^ Humanitarian and Conflict Response Institute The University of Manchester Manchester UK

**Keywords:** COVID‐19, discourse, Foucault, Governmentality, risk science

## Abstract

COVID‐19 demonstrated the complex manner in which discourses from risk science are manipulated to legitimize government action. We use Foucault's theory of Governmentality to explore how a risk science discourse shaped national and local government action during COVID‐19. We theorize how national government policymakers and local government risk managers were objectified by (and subjectified themselves to) risk science models, results, and discourses. From this theoretical position we analyze a dataset, including observations of risk science discourse and 22 qualitative interviews, to understand the challenges that national government policymakers, risk scientists, and local government risk managers faced during COVID‐19. Findings from our Foucauldian discourse analysis show how, through power and knowledge, competing discourses emerge in a situation that was disturbed by uncertainty—which created disturbed senders (policymakers and risk scientists) and disturbed receivers (risk managers) of risk science. First, we explore the interaction between risk science and policymakers, including how the disturbed context enabled policymakers to select discourse from risk science to justify their policies. This showed government's sociopolitical leveraging of scientific power and knowledge by positioning itself as being submissive to “follow the science.” Second, we discuss how risk managers (1) were objectified by the discourse from policymakers that required them to be obedient to risk science, and paradoxically (2) used the disturbed context to justify resisting government objectification through their human agency to subjectify themselves and take action. Using these concepts, we explore the foundation of risk science influence in COVID‐19.

## 1 INTRODUCTION

Before the COVID‐19 pandemic, risk science created knowledge of pandemic risk (e.g., Fischhoff et al., 2018; Wein & Atkinson, 2009) which informed policies/plans to manage a pandemic. In COVID‐19, risk was the combined potential for undesirable consequences of the virus and the uncertainties in the measures taken to control it (Aven, 2022). Risk science was a term used to explain a broad range of risk assessments that were undertaken to provide information on risk characteristics and informed a basis on which to select solutions and precautionary measures to manage that virus (Aven & Thekdi, 2021). Risk scientists did not take the decisions, but their findings informed advice that helped policymakers to understand and measure how risks changed (ibid). To inform decisions, risk scientists developed risk models as decision support tools (Aven, 2015) and local government risk managers acted according to their plans and the knowledge created from analyzing emerging risks (Vekaria et al., 2021) and response options (Siegrist et al., 2021). But, despite claims of the COVID‐19 response being science‐led, Aven and Bouder (2020) suggested it was informed by risk science that was reinterpreted by government to create a political discourse. To explore their suggestion, we analyze the political leveraging of knowledge from risk science to justify government action during COVID‐19.

How risk science influences action is foundational (Aven, 2019) but has not been explored before using Foucault's concept of Governmentality and theory of discourse (Foucault, 1980, 1982, 1984, 1994, 2010). We select Foucault because of his renowned work on Governmentality (Althaus, 2005; Raffnsøe et al., 2019), which is the art of governing through exploiting mechanisms to regulate and control populations, and encourage self‐governance, through the power and knowledge of discourse (Turner, 1997: xiii). Foucault (1972) described discourse as “the interplay of the rules that make possible the appearance of objects during a given period of time” (p.33) and as “tactical elements or blocks operating in the field of force relations” (Foucault, 1984, p.101–p.102) where “points of resistance will open up” (ibid, p.95). As Mingers and Wilcocks (2004) suggested, Foucault's views discourse as an argumentative force for emancipation which involves a process of “unfreezing” people minds to bring about change. For Foucault, discourse is a way of thinking and communicating that is expressed through social interaction and includes talking/writing, and structuring/presenting knowledge as a way to gain or maintain power (Fairclough, 1992). Foucault explains how risk is a socially constructed discourse (Alaszewski, 2021) which governments use to control citizens (Ahrens & Ferry, 2021).

In Governmentality, Foucault explains that scientific discourse is an “indirect mechanism of state power” (Latour, 1987, p.219) which supports governing by “action at a distance” (Latour, 1987) in providing tactics to exercise political power. For example, through Governmentality in COVID‐19, UK politicians claimed submission to “follow‐the‐science” suggesting that policies to control citizen behavior were determined by science (Rydin, 2021). Risk science, then, was a mechanism of political power to influence an “obedient citizen” (Newman, 2022)—a caricature that explains the practice of objectification. In *objectification*, humans are objects of discourse that absorb pressure to be obedient through their submission to it (Heller, 1996). Foucault (2010) argued that governments objectify citizens by positioning scientific discourse as holding an expert's power of persuasion and an expert's knowledge of a well‐informed position. Both power and knowledge must be present for a discourse to have influence so they are linked as the “power/knowledge” of a discourse (Mills, 2003). Dominant discourses hold the most power/knowledge to shape action but alternatives exist through which people can subjectify themselves (Alaszewski, 2021). In *subjectification*, humans resist objectification and act on alternative discourses so “are not just passive victims of the power of strategic discourse, rather they are constituted as subjects either in support of, or in resistance to, its plausibility” (Knights & Morgan, 1991, p.269).

To explain, local government risk managers assess available discourses to justify their action to manage risk (Althaus, 2005). Acceptance of a discourse involves their obedience/submission to it through subsequent actions, thereby objectifying them (Clegg, 1998). However, risk managers are not always obedient (Mills, 2003) as their own discourses of power/knowledge encourages them to resist (Foucault, 1984). Resistance can be based on knowledge of situational uncertainties/disturbances (Alaszewski, 2021) that obfuscate the dominant discourse so both risk scientists (as senders of risk science discourses) and risk managers (as receivers) are disturbed by changing conditions (Neumann, 2008). COVID‐19 witnessed risk managers in a transformational process (Fairclough, 1992) from being objectified to becoming subjectified that Foucault labels *objectified‐subjectification* (Heller, 1996). Our article explores how objectified‐subjectification happened, that is, how government manipulated COVID‐19 risk science to create discourse that held power/knowledge to create obedient risk scientists and risk managers, and how both groups applied their knowledge of a disturbed context to resist and justify alternative action.

To highlight our thesis in a different context, on 14 June 2017 72 people lost their lives in a high‐rise building fire at Grenfell Tower, UK. Risk science developed models (in combination with other disciplines such as engineering) that informed a scientific discourse that the safest action for residents in high‐rise building fires was to “stay put” in their flats. This discourse, with others, informed a policy (a discourse that was dominant) on managing high‐rise building fires. Risk managers were objectified by that policy, were submissive to it, so obediently advised residents of Grenfell Tower to “stay‐put.” As the fire continued, managers were disturbed by emerging information which challenged the discourse. Almost 2 hours after the fire begun, risk managers subjectified themselves to the discourse being situationally flawed and resisted it by transforming their discourse into advice to “evacuate.”

To develop our theory of how risk science informed government action in COVID‐19, we use a qualitative dataset of secondary data of discursive events involving policymakers and risk scientists; 22 qualitative interviews with risk managers; observations made during extensive participation with government. These are analyzed together using Foucauldian discourse analysis (FDA) to understand “forms of practice that are constitutive of the subject and objects that make up the world defined within particular truth regimes” (Hammersley, 2006, p.3). Our findings inform risk scientists of how risk analysis can establish a scientific discourse that influences government action (Dunn & Neumann, 2016). Also, our findings show how objectified‐subjectification explains how human agency can positively transform discourse through resistance.

To arrive at these findings we review the literature on relevant Foucauldian philosophy. We then present the data that informs this study and our use of FDA (Khan & MacEachen, 2021). We present our findings and discuss how these are foundational for risk science.

## 2 LITERATURE REVIEW

Given that risk science has not before been integrated with Foucault's theory of Governmentality, we now summarize his relevant concepts including: power/knowledge of scientific discourses; influencing an obedient citizen; resistance to dominant discourse; transforming discourse; and action.

### 2.1 Foucauldian discourse: power, knowledge, objectification, and subjectification

In COVID‐19, governments presented their discourse as “following the science” (Coleman & Sim, 2021; Evans, 2022) which objectified science and presented it as a single truth to which they were submissive. Aven and Bouder (2020) referred to this as government's manipulation of science, arguing that governments selected COVID‐19 risk science evidence that supported their discourses while ignoring science that contradicted their position. Such behavior is an example of Foucault's theory of Governmentality in action.

Foucault's early work presents humans as objectified products of discourses (Alvesson & Karreman, 2000) which aim to pressure them into a particular response. Townley (1998) suggested that Foucault's citizens are objectively managed by the discourse which is strengthened by their trust in the power/knowledge of argument (e.g., risk managers being objectified by the power/knowledge of risk science findings). This perspective was critiqued as being limited because it is only concerned with creating submissive objects through a dominant discourse (Newton, 1998), whilst actually humans are active agents.

Responding to this critique, Foucault's later work includes human agency through the process of subjectification (Heller, 1996). This involves knowledge and awareness of alternative discourses which prompt humans’ resistance through power/knowledge to leverage their own agency to transform the discourse, to justify acting differently. Fairclough (1992) explained subjectification as a socially interactive process and, from a Foucauldian perspective, is a feature of emancipation (Foucault, 2010). Clegg (1998) explained how subjectification works by acquiring new knowledge, which concurrently create, and is shaped by, alternative discourses from which they construct new meaning (Clegg & Dunkerley, 1980; Foucault, 1982). Clegg (2013) added that the power to resist a discourse increases through collective human agency which enables collective resistance for risk managers during COVID‐19 (Kutay, 2021).

Foucault's theory of Governmentality is exemplified in COVID‐19 where governments used risk science to justify decisions and develop a discourse to manage citizen obedience (Kritzinger et al., 2021), but those discourses did not always reflect risk science. For example, in Italy, there was no description of the characteristics of the deceased early on, so the lockdown policy was decided “without the support of official, real‐time data being available for the public on key surveillance indicators” (Lazzerini & Putoto, 2020, p.641). By lacking evidentiary underpinning, the policy's fragility was exposed, the public resisted, and government penalized non‐compliant behavior such as public demonstrations (Chamola et al., 2020; Martins et al., 2021). Nevertheless, trust in political leadership remained steady (Altiparmakis et al., 2021) as a feature of the obedient citizen.

Lack of trust and resistance to the dominant discourse challenges citizens, creates conflicts, and initiates change. Here, *challenge* is the initial identification of differences in perspective and realizing the potential need to be open to alternatives. *Conflict* escalates challenge through “a set of conflicting discursive frameworks” (Mills, 2003, p.63) that demands the need for rethink. Change is the outcome of conflict which “leads to alterations in trends of thinking and operating” (Downing, 2008, p.15). Challenge, conflict, and change are features of discourse transformation that are used by risk scientists and governments to encourage obedience, and by risk managers and citizens to resist and subjectify themselves (Smart, 2002).

### 2.2 Risk science discourse in COVID‐19 and government action

In COVID‐19, risk science informed new discourses that influenced policy and practice. A *transformed discourse* (Fairclough, 2005) occurred when human agency resisted existing discourse and, by repositioning a discourse through their power/knowledge, embedded a new discourse through action (Heller, 1996). Transforming risk discourses with new scientific insights was a complex process, made more challenging by the need for scientists to negotiate shared mental models to agree assumptions (Nyame‐Asiamah et al., 2023).

This was apparent early in the pandemic when risk science was limited by the lack of data; nevertheless it transformed discourses by providing knowledge on the interaction of complexities. Those discourses held power/knowledge from a risk science process (Thekdi & Aven, 2023), that is, used “all knowledge, founded on data, information, models, tests, analysis and argumentation” and used probabilities to express uncertainties despite their limitations (Aven & Bouder, 2020, p.850). These limitations were underplayed in policymakers’ discourse which, instead, reiterated the need to trust scientists and, by association, trust science‐led government action (Algan et al., 2021).

Such influence was not new to risk analysis as, when results have power, their knowledge transforms discourses and drives action (Thekdi & Aven, 2023), for example, established new responses and policy in COVID‐19 (Alaszewski, 2021; Tierney, 2012). But, Foucault (1982) advised that “power only exists when it is put into action”, so power/knowledge and action are inseparable. Furthermore, the power of risk science can be lost when its knowledge (e.g., assumptions) is undermined or manipulated (Kutay, 2021). Such scenarios created space for transforming discourses so give risk managers opportunities to resist a scientific discourse in preference for alternative/stronger discourses that compete (Lazzerini & Puloto, 2020).

### 2.3 Research questions

In this context, our research questions explore how risk science shaped government action in COVID‐19:

1. How did risk science discourse influence UK government action to manage COVID‐19 risk?
2. How did UK risk managers decide between competing discourses when deciding on action in COVID‐19?

## 3 METHODOLOGY

### 3.1 Context and philosophy

Our study is underpinned by the philosophy of constructionism (Miller, 2008) which asserts that multiple meanings of truth and human activity are created through social interactions (Gergen, 1985; Velody & Williams, 1998). In the COVID‐19 context, the power/knowledge held by risk scientists (and others) varied which meant there were multiple truths to study and these were made transparent through the different discourses available. These discourses were supported by risk science models and were formed by the social interaction between different groups that sought to influence policy and shape government action (Marchesi & Luigi, 2022). We analyzed these discourses using FDA (Arribas‐Ayllon & Walkerdine, 2008).

Scholars who are interested in Foucault's theories come from a wide range of disciplines (Fairclough, 2013) which bring a philosophical and methodological diversity to Foucauldian research (Hall, 2005). However, a constant presence in empirical studies that take a Foucauldian lens is discourse analysis, often labeling this FDA (Khan & MacEachen, 2021; Sam, 2019). The *Foucauldian* part of this label relates to the lens applied to the analysis of discourse as a political and social interaction as provided by Foucault's philosophy and related theory (Riley et al., 2021). As a critical theorist, the *Foucauldian* part of the label also points to critical nature of analysis related to “connections between the use of language and the exercise of power are often not clear to people, yet appear on closer examination to be vitally important to the workings of power” (Thompson, 2004, p.5, quoting Fairclough). The *Discourse Analysis* part of this label relates to studying “texts [that] constitute a major source of evidence for grounding claims about social structures, relations, and processes” (Fairclough, 1995, p.209).

As a philosopher, Foucault did not develop a methodological approach (Graham, 2010) so there are deviations from the FDA labeling. Some scholars label their approach “Foucauldian Critical Discourse Analysis” (FCDA) (Jäger & Maier, 2009; Tang et al., 2022) to reflect that Foucault's work was explicitly shackled to critical theory (Wandel, 2001) as “one of the theoretical ‘godfathers’ of CDA [Critical Discourse Analysis]” (Wodak & Meyer, 2009, p.10). However, FCDA is an acronym shared with Feminist CDA (Jan & Rahman, 2022). Furthermore, Riley et al. (2021, p.285) asserted that “Foucauldian‐informed discourse analysis [is] (also known as poststructuralist discourse analysis).” Key to whichever label is used is the unique sociopolitical approach to analyzing discourse in Foucauldian studies—for example, that the linguistic approach of “discourse as language” is rejected to focus on a more social interpretation of “discourse as a system that involves social interaction” (Hall, 2005; Wodak & Meyer, 2009).

We align with discourse as a system of social interaction and apply FDA to explore how power/knowledge circulates between a system of actors interacting on risk science. This means that discourses are not merely exercised top‐down but that other dynamics must be studied to understand how discourses circulate, as “discourses are practices and are therefore mobile, flexible, instantiated differently in different contents and always transformational” (Wetherell et al., 2005, p.194).

To analyze these circulating discourses in COVID‐19, FDA enabled us study “discourses that a participant uses when making sense of an issue … [those] circulating in public enough to structure thought” (Riley et al., 2021, p.294). Analysis across interviewees enabled us to identify commonalities and differences (i.e., how contested domains of meaning influence alternative discourses). Our findings show how human agency and social relations are reconstructed through power/knowledge and action.

### 3.2 Data sources

To answer our research questions within an FDA approach we collected discursive data from three actors: national government **policymakers**, **risk scientists**, and local government **risk managers**. Table 1 shows the diversity of secondary and primary data, including participant observation, videos, documents, and interviews. Analyzing discourse through a Foucauldian approach permits the use of multiple data sources to understand their content (Mohammed, 2021) so we sourced secondary and primary data to explore the challenge, conflict, and change present in COVID‐19 discourses from our three actors. The analysis of secondary data is an important feature of FDA (Boulton et al., 2022). Our data collection was conducted in two phases and virtually due to UK lockdown restrictions.

**TABLE 1:** Sources of data used in our DA to identify transformed discourses

	Data source		Risk scientists	Risk managers
Phase 1	Participation	Participant observation	✓	✓
		Informal conversations	✓	✓
		Presentations, system designs	✓	✓
		Feedback on work produced		✓
		Field diaries and weekly de‐briefs		✓
Phase 2	Videos	National briefings to the public	✓	
		In‐person evidence to government committees	✓	
		BBC documentary: *Lockdown 1.0*	✓	
		News programs	✓	
	Documents	Government guidance		
		Minutes from SAGE	✓	
		Written submissions to government inquiry	✓	
		Published reports	✓	✓
		Newspaper articles	✓	
		Websites	✓	✓
		Blogs and social media posts	✓	
		Written case study of activities		✓
	22 interviews			✓

**Phase 1** focused on the context of discourses by capturing discursive preconditions of COVID‐19 from our three actors as well as discourse challenges from scientists, government, and nongovernmental organizations. Two researchers actively supported national and local government committees by contributing to, and observing, 1500+ hours of meetings from March 2020 to December 2022. Nationally, we held informal discussions with policymakers and risk scientists, including from the national government‐appointed Scientific Advisory Group for Emergencies (SAGE) that provided risk analysis during COVID‐19. Locally, we supported risk managers in UK local resilience partnerships that are the multi‐agency groups of statutory agencies for risk and resilience. Here, we were daily embedded with three such partnerships and engaged with over half of the UK's local resilience partnerships through weekly workshops which we ran to support their planning. Across all engagements, we made extensive field diaries of every meeting, activity, participant observation, informal conversation, presentation, system design, and feedback. We conducted weekly de‐briefings to consolidate our learning. Our virtual participation was enduring and immersive through which our understanding of the social construction of power/knowledge and its relationships to action became immeasurably richer.

**Phase 2** included collecting 51 sources of secondary data (see Table 1 and Appendix A) from policymakers and risk scientists that showed their COVID‐19 discourse. Publicly available documents and videos such as the BBC documentary “Lockdown 1.0 – Following the Science?” were collected which gradually exposed differences between our three actors. Primary data was collected through 22 semistructured qualitative interviews with risk managers (see the protocol in Appendix B and interviewees’ role in Appendix C). Interviews are “considered a social situation in which societal discourses are articulated” (Riley et al., 2021, p.294) and provide insight to interpretive repertoires (i.e., patterns of talking, behaviors, and contradictions) that “recognise routines of argument” (Reynolds & Wetherell, 2003, p.496) which illuminated their transformation through objectified‐subjectification. Using Phase 1 insights, interviewees were recruited from UK local risk managers based on their innovative response to being objectified early in COVID‐19. Twenty‐one interviews (averaging 65 min) were recorded (with permission) between October 2021 and April 2022 and transcribed—one interviewee preferred only handwritten notes taken. Ethical approval for was granted by the University.

### 3.3 Data analysis

Parker (2014) suggested that analyzing discourse can usefully analyze contextually specific phenomenon where there is conflict and change. Our FDA began by exploring the three core influences of discourse transformation (i.e., challenge, conflict, and change—explained before) that create discursive shifts in actors from objectification to subjectification.

We applied FDA to **Phase 1** data to appreciate how discourses adapted over time (Riley et al., 2021). This involved us collecting data to identify the process from objectification to subjectification. We understood, extracted, and mapped data relating it to three key influences of Foucault's discourse transformation regarding how discourses: (1) ***challenged*** the obedience of risk scientists and risk managers through the lack of data and the rise of multiple competing scientific discourses, which changed their positioning from obedience to argumentative interpretive repertoires; (2) ***conflicted*** the obedience of risk scientists and risk managers to the national policy, encouraging them to eventually reposition their local risk discourse; (3) ***changed*** both risk scientists and risk managers to be subjectified political actors, whereby risk scientists vocalized the manipulation of risk science and risk managers operationalized their local risk knowledge. To accomplish this, we pinpointed discourses that existed in spoken or written data or that we observed in interactions and behaviors. Such use of multiple data sources is commonplace when taking a Foucauldian approach to analyzing discourse (Mohammed, 2021) and in qualitative data analysis more generally (Bryman & Bell, 2015). For example, we observed that risk scientists’ discourse *emerged* as being objectified by government manipulation. Resistance and challenge manifested through scientists contesting that manipulation, and change was evident by them publicly questioning the policy interpretation of science and amplifying alternative science that supported different policies.

In **Phase 2** we used FDA to understand how and why the wider population of risk scientists and risk managers were obedient to policies based on fragile science, identify trigger points of conflict, and change that resisted a manipulated national risk discourse, and manifested “social action and interaction” to transform to being subjectified (Fairclough, 2001, p.123). We amassed evidence of how challenge, conflict, and change influenced discourse. To formalize this process, we worked with all 22 interviews and secondary data to map the discourse through which the emergence of language, meanings, actions became more structured. To explore objectified‐subjectification, we identified how transformed discourses were produced through the power/knowledge of challenging, conflicting, and changing discourses, and their resistance and transformation. For this, we needed rich descriptions from triangulating data, to appreciate the discourse of objectification and subjectification (Johnstone, 2017).

To enhance the rigor of Phase 2 analysis, we followed Guba and Lincoln's (1985) four quality criteria for trustworthiness of qualitative research: First, to ensure the *credibility* of findings the interviewees checked our written summaries of their objectified‐subjectification and validated versions of these were published as case studies (Scully & Shaw, 2022). We conducted methodological triangulation by cross‐referencing emerging discoveries across our Phase 2 data (Jonsen & Jehn, 2009). *Transferability* of findings involved developing thick descriptions of our discoveries and testing their generalizability in analogous contexts (such as the Grenfell Tower fire and Italy's COVID‐19 response). Third, two researchers peer reviewed how our discoveries depicted the reality contained in the data to ensure *dependability* of our findings. Finally, *conformability* that findings are rooted in the reality of research subjects (not researchers) was attained through process‐driven FDA analysis.

**FIGURE 1:**
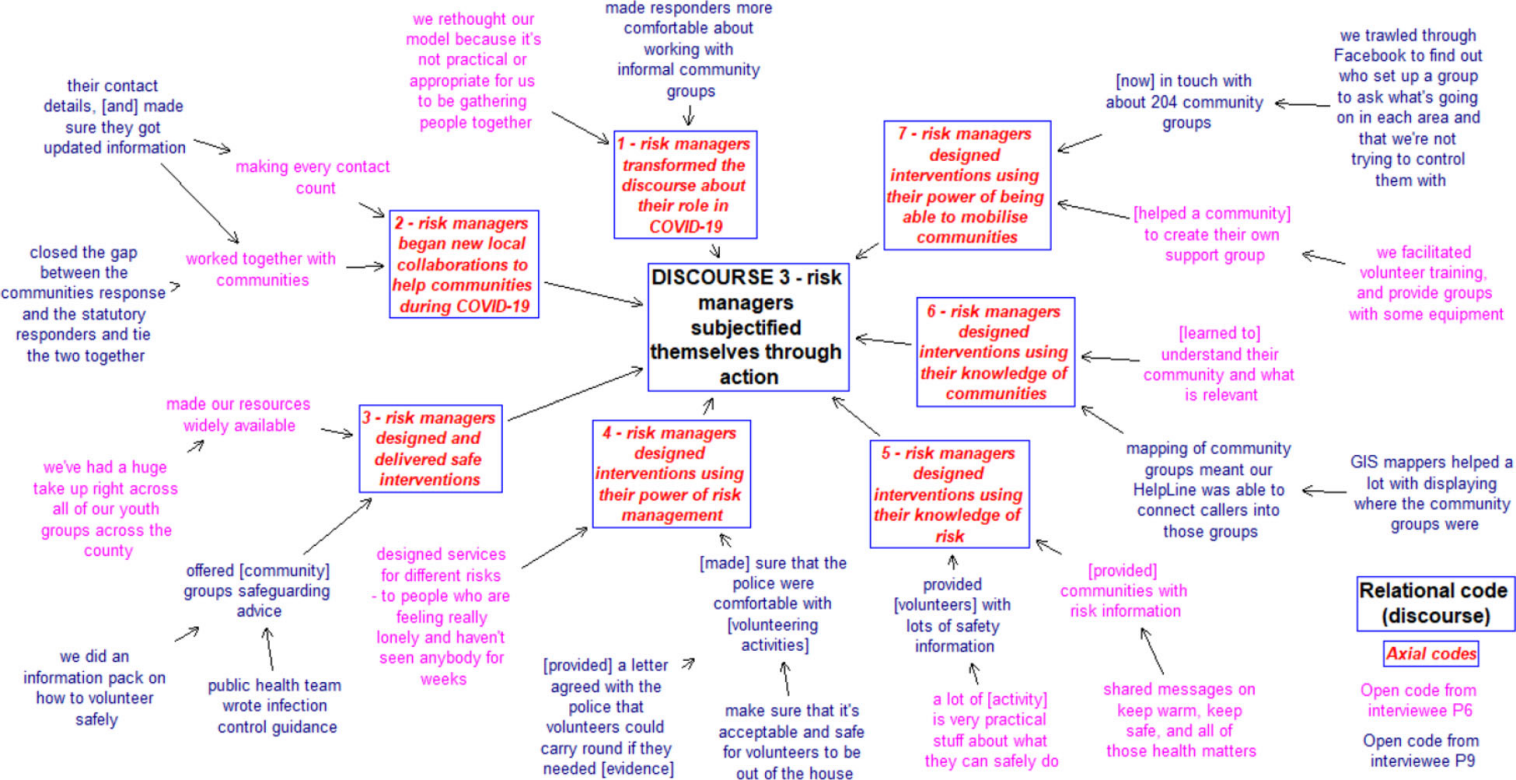
Example of data analysis map for risk managers for Discourse 3: Subjectification through action

Analyzing the data involved a mapping approach that built a Soft Operational Research model (Gomes & Schramm, 2022; Mingers, 2011) to visually represent codes from our primary and secondary data (Smith & Shaw, 2019). The model (called a “map,” see Figure 1) was built through three stages of thematic coding (Braun et al., 2022). First, open coding reduced all the Phase 2 data (all 22 interviews and secondary data) by identifying relevant quotes from audio/text/notes that represented important aspects and placed these as written “concepts” into an emerging map. Then, axial coding was conducted to discover relationships across concepts that were coded as arrows connecting two concepts (A and B) between which there was a hierarchical relationship (in our case, A is part of B). The map's structure emerged by more arrows and concepts being added from different sources—thus the axial importance of concepts emerged through triangulating concepts. One example map is Figure 1 that shows the relational code “subjectification through action” that explains Discourse 3 from risk managers and how that is informed/structured by the content of descriptive axial codes. Two other relational codes (discourses) also emerged, aligned to Foucault's theory of Governmentality, which structure Section 4, namely: Discourse 1 “Risk science discourse dominating despite its fragile basis”; Discourse 2 “Objectifying risk science” (see Table 2).

**TABLE 2:** Discourses from risk scientists and risk managers inspired by challenge, conflict, and change

	Discourse 1: Influence of challenge	Discourse 2: Influence of conflict	Discourse 3: Influence of change
**Discourse from risk scientists**	Risk scientists can develop risk science models to analyze the COVID‐19 pandemic to create advice—but science is challenged because the context is disturbed by competing findings/opinions emerging from different scientific disciplines/groups, and good data is absent	Risk scientists should support government even though risk science models will be in conflict because the power/knowledge of science was manipulated through government directing science and then selectively using scientific findings to justify policy	Risk scientists should change their approach to take political action to make their advice more transparent and raise the profile of different scientific views (e.g., through Independent SAGE), and make citizens aware that science could be made to look like a scapegoat
**Discourse from risk managers**	Risk managers are in a context challenged by uncertainty and risk because: there is little data to inform risk decisions; systems are not fully prepared; national government are making risk‐based decisions that have local impacts—so risk managers should support the national endeavor to keep people safe from risk	Risk managers are in conflict because they should be taking more decisions to manage the risk but have no power/knowledge to do so, and risk managers cannot be seen to resist national government—so will await and accept instruction from national government and then act to manage risk	Risk managers should change their approach to be subjectified through transforming our local action to minimize risk—so Risk managers will transform themselves to design and deliver safe risk‐informed practical action through their power/knowledge of risk and their local communities
**Summary of the discourse**	**Risk science discourse dominating despite its fragile basis**	**Objectifying risk science**	**Subjectification through action**

### 3.4 Illustrating our data analysis

While our analysis mapped relevant quotes from all 22 interviews and secondary data, Figure 1 shows example content as an extract of data from two interviewees (P6 and P9) for Discourse 3 “subjectification through action” in relation to how those two interviewees operationalized their action through subjectifying themselves. Figure 1 is an example that identifies seven axial codes for Discourse 3 (titled 1–7), each detailed with illustrative open codes from risk managers. Figure 1 does not represent all of the open/axial/relational codes from all 22 interviewees and secondary data.

Our findings focus on how risk scientists and risk managers transformed their action through objectified‐subjectification—so our analysis focused on the discourses from risk scientists and risk managers, as detailed in Table 2. Discourses 1–3 are assembled from across different data sources to unify their content related to our three key influences of discourse transformation: challenge, conflict, and change. Our reporting of Discourses 1–3 in Section 4 are structured using the codes found from our coding analysis. To explain, the researcher‐generated axial codes described common characteristics across our open codes, and the researcher‐generated relational codes described common connections across axial codes. Thus, the actors’ discourses that describe the “discursive struggles” (Sharp & Richardson, 2001, p.199) are based on the relational codes and enunciated by the axial codes which provide a structured decomposition of different dimensions of each discourse. The actors’ discourses that describe the discursive backbone of our findings are summarized in Table 2. In Section 4, the axial codes sit in background because these are researcher‐generated, and FDA advises that the actors’ discourses should be foregrounded to expose “the dynamics of conflict and struggle [that] become the centre of analysis” (Flyvbjerg, 2001, p.6). By foregrounding the actors’ discourses from Table 2 we expose the “power struggles between different interests, where knowledge and truth are contested” (Sharp & Richardson, 2001, p.198) and thereby amplify the competing voices of discourse owners. For other examples of FDA in action see Shook et al (2022), MacKenzie and Murray (2021), McDonald et al. (2021), Gumley (2021), Kavoura et al. (2015), MacKay and Zuffrey (2015), Moore and Seu (2010).

## 4 FINDINGS

Foucault's theory of Governmentality and FDA's three key influences of discourse transformation (Potter & Wetherell, 2002) influenced our three discourses from Table 2 that structure our findings. Discourse 1 explains how the risk science discourse emerged as reporting a scientifically based truth but was initially challenged because of flaws in the unstable nature of the modeling and prevalence of competing scientific discourses. Discourse 2 explains how policymakers used that fragile discourse to objectify risk scientists by manipulating scientific findings and objectifying risk managers by expecting obedience to those science‐based instructions. Simultaneously government claimed its own obedience to science in formulating those instructions. Objectification conflicted with scientific independence and risk managers’ subsidiarity principle (that decision‐making should be devolved to the lowest competent level), establishing the basis for their resistance. Discourse 3 reports how risk scientists and risk managers resisted objectification by subjectifying themselves to change their discourse to justify their alternative action.

S1–S51 refers to secondary data sources (see Appendix A); scientists from BBC documentary S1 are cited as “S1:Surname of scientist”; and P1–22 refer to interview participants (see Appendix C).

### 4.1 Risk science discourse dominating despite its fragile basis

Our observational fieldnotes (from 28 April 2020) included how **risk scientists** established a discourse that “**scientific knowledge can advise on how different controls can limit the impact of COVID‐19**” which supported policymakers’ discourse of “follow the science” (e.g., S1, S22, S24, S33, and S41). This was stated early in the pandemic:

As a country we are in the fortunate position of being home to an extraordinary wealth of experts – scientists, researchers and academics – all at the forefront of their chosen fields … dedicated their whole lives to studying, researching, and honing their knowledge … expertise, including in vaccines and pandemics; knowledge that is now being applied to tackling COVID‐19. …. We need these experts more than ever … our approach to this virus will always be guided by the scientific evidence. … Together, we all need to … follow the science (S22).

However, risks scientists were challenged to deliver on this “in the first weeks of the UK's epidemic, [as] it was difficult for SAGE to accurately assess the state and trajectory of the outbreak at that time due to the lack of data …[because] not all [SAGE] modelling groups were able to access [healthcare data] initially” (S23) leaving some to “us[e] Wikipedia to get data very early on in the pandemic, but that was really the only data [available]” (S1:Hall). To increase the model accuracy, government bodies pooled data to “provide a single source of truth about the rapidly evolving situation” (S48), and SAGE contrasted results from “at least three different, independent [models] … to generate a consensus view” (S1:Medley). Initially, minutes from SAGE meetings reflect “considerable uncertainty around the data … a reasonable worst case cannot be made reliably under such circumstances” (S48) signaling “considerable uncertainty around estimates” (S4). Even as late as August 2020, scientific methodologies were still challenged as the government “chang[ed] [its] approach to reporting deaths” (S6).

Through leaks we learned of “concerns that [SAGE's scientific] independence has been compromised” (S7) by political advisors attending meetings. Risk advice from important disciplines were under‐represented such as “business … engineering … logistics … virology … immunology” (S8). Other actors were also challenged to have their risks heard, for example, care home staff's “lobbying around trying to get testing… [but government said] there was no evidence that routing testing was a worthwhile approach” (S1:Monaghan). Challenges to the science emerged: “data scientists made the nearest thing to an unequivocal error with the modelling they did around the risk in care homes, by failing to understand the movement of staff between homes” (S2). Despite these fragilities, risk science continued to provide power/knowledge to policymakers’ discourse to “give policy advice … based on a broad set of scientific principles” (S9).

Local government **risk managers**, according our fieldnotes (from 13 April 2020), developed a risk management discourse: “**we will manage our local risk to keep residents safe [in COVID‐19]”**. Safety included “opening peoples’ horizons and making them aware of the risks” (P6), “[ensuring] PPE for our own safe operations” (P12), and “providing online safety training for volunteers” (P4). From our participation in a national COVID‐19 committee, three workshops (each with >160 risk managers) showed a challenged risk science discourse which changed over 6 months (May–October 2020): Initially the “strongest” risk manager discourse was their challenge of having no local risk science because “national [government] are not sharing data, modelling, information, strategy, decisions or knowledge” (S49), a concern mirrored in the second workshop (S50). By the third workshop, this discourse was partly transformed to an “increasingly improving picture of information and data exchange at the local level” (S51). As risk managers had insufficient power to influence national practices, their discourse of “[having positive] impact on the person I'm trying to help” (P20) continued.

### 4.2 Objectifying risk science

Objectification of **risk scientists** took many forms: First, policymakers heralded risk science as giving them mastery over COVID‐19 and justified their actions. For example, they stated “we are committed to doing everything possible based on the advice of our world‐leading scientific experts” (S10) and “are following the science and introducing public health measures that are supported by SAGE” (S33). But policymakers did not follow all the science as it manipulated conflicting advice that suited their political priorities by “taking advice from a large number of people” (S1: Medley).

To exemplify the manipulation that arose from conflict, on 22 December 2020, SAGE advised that controlling the virus was “highly unlikely… with schools open … to maintain R below 1” and “R would be lower with schools closed” (S6), with R being the reproduction rate of the virus. On 3 January 2021, policymakers insisted schools will restart as “the evidence is not clear… scientific advisors have said different things at different times, they are by no means unanimous … schools are safe” (S20). The following day, policymakers “pushed ahead with the opening of most primary schools in England” (S19) but in a U‐Turn, at 8 p.m. that day, announced that schools “must move to remote provision [shut immediately]” (S35) due to ongoing concerns. Other examples emerged: “Government have flatly contradicted the Covid guidance from one of their most senior health officials that people should not socialise before Christmas… our most senior adviser gave advice that was completely denied by No. 10 [the Prime Minister's Office] and the Government” (S46). Risk scientists were objectified as having mastery but then manipulated when policymakers preferred a different discourse.

Policymakers promoted scientific advice as reporting a single truth: “it is convenient for government to feed into this idea that scientists can deliver the [COVID‐19] science as some sort of absolute truth … because then it gives politicians’ decisions some sort of legitimacy—but that's not really true, is it?” (S24). A government advisor agreed, “It's very much not, uncertainty is the real thing here … it was all about trying to express the uncertainty and the boundaries of that … explaining what you know and don't know” (S24). Despite this, we found no examples of policymakers’ discourse revealing either uncertainty, being a disturbed sender of selected advice, or that advice being from a disturbed context, prompting a SAGE scientist to express “there is going to be a lot of criticism of the scientists” (S1: Semple) as the conflict in the science was not transparent. Instead, policymakers projected a discourse that supported government's political action justified by manipulated science. The justification was regularly announced by government ministers in national televised briefings (S37) when policymakers were normally flanked by the Chief Medical Officer and Chief Scientific Officer, who were highly regarded figures with considerable power/knowledge (S11, S12). These figures further heightened the power/knowledge of policymakers’ discourse to encourage viewer obedience.

Scientists were objectified to maintain public confidence, for example, by legitimizing policymakers’ actions, as one SAGE scientist said “we need to keep trust in the leadership, in the government, because that is what is needed in order to manage this pandemic” (S1:Michie)—conflicting with their scientific independence. SAGE scientists also heightened their own status by claiming “extremely good modelling” (S45) and “we were in quite a good position to understand what might happen in the United Kingdom” (SL1: Medley).

Initially **risk managers** were objectified by policymakers’ discourse that “[national] government need to actually consult about what is planned, not simply announce something at a press conference and expect [us to make] it to work the next day” (S50). For example, policymakers announced that risk managers “will create a network of local hubs … [to deliver] medicines, groceries and essential household items … [involving] volunteers … everybody can contribute in different ways” (S31). Some risk managers were conflicted, feeling that unsupervised volunteers should not work in hazardous situations with vulnerable people (S15, S16) but had little alternative other than manage risks from the “potential for the spread of the virus” (P17). Further objectification was by policymakers withholding national resources, for example, the health service had “400,000 ‘on duty’ volunteers … [which risk managers] tried to use … and just couldn't get any access to” (P10).

Risk managers accepted policymakers’ instruction and focused on delivering risk management professionally to a response being “run by national government” (P13). Risk managers were conflicted by having to “build and fly the helicopter at the same time” (P11), having to react “very quickly around what the need was, what we could do to support that” (P16) despite the risks. Objectification also took the form of withholding data from risk managers so managing the local response was hindered as “information sharing is woefully lacking from [national] government … [and] the lack of clear data [from policy] to inform our planning has been less than helpful” (S50).

### 4.3 Subjectification through action

Risk scientists and risk managers subjectified themselves through their human agency of power/knowledge to resist objectification and change their discourse.

This began when some **risk scientists** “started to think that things were really not in control” (S1: Davies) beginning an unease about not *following the science*. To enhance scientific scrutiny and balance, risk scientists changed approaches to “putting scientific facts and debate into the public domain” (S17) though founding “Independent SAGE” (indie_SAGE). Indie_SAGE quickly established its power/knowledge as “independent of government … open and transparent” (S17). This changed discourse continued as SAGE and indie_SAGE scientists exposed the modeling challenges and troubled relationship between science and policy (S1). For example, following her initial desired to “keep trust in the leadership” (S1: Michie), the SAGE advisor talked about her later subjectification: “Had I known what I now know I would have been much clearer and said we should be shutting down all gatherings now” (S1: Michie). Meanwhile, advisors changed discourse to admit that scientific modeling may have been too influential as “[government got] into a mess by relying on modelling and allowing modelling to drive the whole response. And the failure of science … will be seen as one of the most important features of what has been a very, very poor response” (S1: Scally). Subjectification also involved scientists questioning whether scientific risk modeling was influential: “‘following the science’ is a meaningless phrase. I don't believe any government follow the science, nor should they. It seemed to quite a few of us [scientists] that this may be more to do with PR [Public Relations] and, potentially, scapegoating scientists” (S1: Michie).

**Risk managers** subjectified themselves by changing their discourse to justify their innovative action. This enabled risk managers to transform the discourse about their role in COVID‐19 (axial code 1 in Figure 1) which began with their changed discourse that policymaker instructions were *chaotic at times* (P3) and lacked risk and safety consideration, so they had to supersede that with their own creative provision. For example, “national government announced that councils would have to run a helpline” (P9) which risk managers were not ready to do as “most of [our volunteers] had no training at all … [so] groups linked to that helpline, where the volunteers wouldn't have had any formal training” (P9). Subjectification enabled risk managers to form their own discourse to “manage our local risk to keep residents safe” (P10), so quickly built solutions that began new local collaborations to help communities (axial code 2) centered around “community hubs” (P6), “mobiliz[ing] existing relationships” (P21), and creating new systems “like community Uber for volunteers” (P11)—but others resisted “no, not doing it … it's too risky” (P10). Thus, risk managers were subjectified to deliver safe interventions (axial code 3) to manage risk (e.g., “nobody was to travel in cars or taxis … [as] it wasn't safe” (P17), and “get data protection right” (P10)).

Our fieldnotes show that risk managers felt that policymakers heightened the *illusion of being in control of the crisis* (29 April 2020) but their lack of local knowledge and risk awareness was a catalyst for resistance. For example, risk managers changed their action by resisting national government's “atrocious [food] supplies … not good enough [so] how do we begin to supplement and develop that” (P2) with a more culturally appropriate offering. Risk managers’ resisted instructions that conflicted with the “priority of volunteers and public safety” (P22) and transformed those instructions to “creative solutions” (P20) that “rapidly targeted at‐risk groups” (P1). These are examples of designing interventions using their power of risk management (axial code 4) by “horizon scanning and looking out for new risks” (P6) which helped “responders to be more comfortable” (P9).

Subjectification freed risk managers to innovate change out of a “completely different situation … everybody was scratching heads” (P11). They used their knowledge of risk (axial code 5) to understand “health and safety guidance around COVID‐19” (P18) to keep their staff/volunteers safe, “conduct risk assessment for volunteers” (P4), and provided “risk awareness training” (P5) and advice on “safety issues” (P16). Their knowledge of risk was enhanced by their commissioning of local risk science to create local COVID‐19 datasets that track conditions in a “data dashboard” (P16) and used “GIS mapping” (P14) to refine local intelligence to change local decisions. Key to subjectification was being “allowed to make decisions at speed … because I knew exactly the rules within which we were working … not being afraid to make the decision and implement it” (P2) and also resisting unacceptable risks such as from “spontaneous volunteers that just turn up create a risk dilemma” (P7). Subjectification enabled them to use their knowledge of communities (axial code 6) garnered from extensive partnership working to “understanding each others’ needs and build trust” (P20) in a “consultative and collaborative [manner] and when it didn't work we still got on with it” (P2). By working with new partners, risk managers used their power of being able to mobilize communities (axial code 7) including “volunteers [with] valuable knowledge of their local area” (P7), “business” (P5), “business networks” (P4), and groups on “Facebook” (P9). New functions included a “help line” (P10), “safe transportation” (P17), and “mass surge testing” (P4). Extending existing functions included “running seven days a week” (P7) and “working … [in] the most culturally and religiously appropriate ways” (P19).

## 5 DISCUSSION

Our findings explore how risk discourses from policymakers, risk scientists, and risk managers interplay in the COVID‐19 response. We now discuss our research questions.

On *how did risk science discourse influence UK government action to manage COVID‐19 risk (Question 1)*, our findings provide insight to Governmentality by illustrating how policymakers claimed to be influenced by the COVID‐19 risk science discourse and used it to justify their action. However, the situation is more complex as policymakers positioned risk science as independent of government, and positioned itself as being objectified by risk science, so took action that obediently “followed the science.” The art of Governmentality (Lemke, 2002) in our context, exemplifies how policymakers selected parts from the risk science discourse, transformed those to support their action, realigned those to risk science, and positioned the risk science power/knowledge as the reason for action. Thus, risk science was tailored as a political tool selected from scientists who had credibility and then manipulated by government (Gane & Johnson, 2013). The art of Governmentality continues by using risk science power/knowledge to objectify receivers (predominantly risk managers in our case), requiring them to be obedient to the scientific power/knowledge. This initially compelled risk scientists and risk managers to follow policymakers’ instruction even where it challenged their risk knowledge.

In COVID‐19, risk managers were disturbed receivers of discourse from disturbed senders (e.g., policymakers and risk scientists) in a disturbed system. A disturbed system creates uncertainty so no single truth emerges, which provides space for objectified actors to harness challenge, conflict, and change to mobilize their own power/knowledge and human agency over what dominant discourse shapes their action (Raaper, 2016). Through this transformation process, which Foucault (2010) viewed as objectified‐subjectification, risk managers create space to consider alternative discourses.

Our findings show resistance from risk managers to being objectified by a risk science discourse (Raffnsøe et al., 2019). Their resistance involved them making choices on how to manage risk and transforming onto alternative discourses (Aven & Bouder, 2020). Those opportunities for transformation arose from the uncertainty of the situation and their contextual knowledge. For example, in COVID‐19, policymakers foisted the concept of involving volunteers onto risk managers, some of whom had long‐held discourses of risk and cautioned volunteers’ involvement (Harris et al., 2016). Risk managers were, thus, disturbed receivers of scientific discourse (Thekdi & Aven, 2023) who confronted the disturbed system by innovating to involve volunteers. To accomplish this, risk managers used their human agency to decide action that aligned with their risk management perspective.

This brings us to how risk scientists and risk managers can shape government action through establishing their alternative discourse. Here, Morgan's (2006) analogy of *psychic prison* is useful—when people are unable to consider alternatives because they are imprisoned by a dominant discourse. To be open to alternative discourses, prisoners must realize the limitation of the dominant discourse with urgency for an alternative. This is possible when the dominant discourse is known to be failing, such as in the Grenfell Tower example presented earlier (MacLeod, 2018). Failing discourses facilitate the space for alternative discourses from risk science to inform disturbed receivers (e.g., risk managers) who become emboldened by their mastery of alternative discourses and their understanding of uncertainty, strengthened by their agency for change through their subjectification (Fairclough, 1992).

On *how did UK risk managers decide between competing discourses when deciding on action in COVID‐19 (Question 2)*, we found that risk managers exercised their subjectification through the transformational process of changing discourse (Fairclough, 1992). This involved the interplay between the challenge and disturbance to a risk manager's mastery of their local knowledge and their resistance to the prevailing discourse. Our findings show how objectified‐subjectification was evident in risk managers’ practice during COVID‐19 and explains how they resisted objectification by mobilizing their human agency to transform the discourse to justify their different course of action (Foucault, 2010).

Discourses on risk were constantly challenged by a disturbed system as well as by alternative discourses (e.g., from citizens) that contained suggestions to manage risk (Kutay, 2021). For example, Aven and Bouder (2020) advised that public knowledge was an important source for risk analysis in COVID‐19 that brought clarity to how the public would react to risk management controls. In many countries the public were mostly obedient citizens during COVID‐19, but this obedience transformed to discontent when they lost trust in the government's pandemic leadership. In the United Kingdom, a discourse transformation occurred when key government figures were exposed to have breached early lockdowns, such as Robert Jenrick (April 2020) and Dominic Cummings (May 2020). Such revelations fuelled public distrust in government (Davies et al., 2021) resulting in a discourse of falling confidence in politicians (Fancourt, 2020), eventually gaining momentum to oust the leadership.

On the politicization of scientific discourse during COVID‐19, Aven and Bouder (2020) affirms that risk science was diluted for political gain and risk scientists resisted this by amplifying their discourse through alternative expert groups and media which, in the United Kingdom, included Independent SAGE. Such politicization of scientific discourse is not new, for example, around the advice from Italy's National Commission for the Forecast and Prevention of Major Risks following the 2009 L'Aquila earthquake (Thekdi & Aven, 2023). The prosecution of seven Commission members claimed that their scientific discourse led citizens to act in ways that put them in greater peril (Alexander, 2014). The scientific discourse was deemed to be “superficial, approximate and generic” and members “failed to carry out their legally binding duties as public officials” (Cartlidge, 2014). Almost in contrast, BBC's *Lockdown 1.0* saw risk scientists subjectify themselves to expose the fragile state of their early scientific results and advice, suggesting the early policy basis was superficial. Overcoming superficiality requires risk scientists to be aware of other risk knowledge such as, in our data, how care‐homes interact with communities and lags in health data. Other knowledge provides risk scientists with mastery of the context (Davies, 2006) and the ability to make their models more comprehensive, and useful.

Our article shows how Foucault's Governmentality offers a framework relevant to risk analysis, risk scientists, and risk managers, which helps explain their experience of COVID‐19. Power and knowledge of disciplines, roles, and individuals are integral to theories of discourse (Foucault, 2010) and Governmentality (Mills, 2003). Foucauldian theory highlights a fragility of all types of scientific discourse which, according to Wetherell and Potter (1988), is related to how scientific discourse is constructed from a combination of empiricist and discursive interpretive repertoires. Methodologically, FDA allowed us to explore contested domains and change through the paradox of obedience and resistance, which are also viewed as political forces (Coleman & Sim, 2000). Practically Foucault (2010) pointed out how resistance and subjectification can be positive features of emancipation and reminds us of the importance of the challenge, conflict, and change of pre‐discursive knowledge.

## 6 CONCLUSION

Our article raises practical issues concerning the precarious aspects of sociopolitical discourse on risk. In COVID‐19, risk science developed new models to provide scientific evidence, addressing a vacuum created by the impact of uncertainties and disturbances (Aven & Bouder, 2020). Filling this vacuum enabled OR science discourses to inform a social process of conceptualizing COVID‐19, but this sense‐making process was complicated by uncertainties which OR attempted to understand, model, and advise against. The uncertainties undermined risk modeling as data was not available and the context not well understood, and compromised the social process of sense‐making as alternative discourses were suppressed or proliferated (Picione et al., 2021). Thus, risk science results were challenged by alternative discourses that competed for influence.

To have their alternative discourses heard, risk scientists may need to build their own power/knowledge with policymakers and risk managers to become trusted sources of discourse. This also involves communicating their alternative discourse in a way that is understandable to policymakers and risk managers who may not have a risk science background. The contribution of this article to risk scientists and risk managers is in providing Foucault's theory and concepts to explain how scientific discourses can establish accepted understanding of the social world where constructed meaning and multiple truths of disturbed contexts are used to justify government action. Considering the power/knowledge held by competing discourses (even when those discourses are thought to be fragile) reminds risk scientists of the need to balance knowledge with power as they are inseparable when seeking to influence action. COVID‐19 saw a proliferation of scientists amplifying their discourses through blogs, media, providing advice to government panels and proffering alternative solutions in the disturbed setting where traction was hard to garner. Publishing scholarly work is one way to influence a discourse, and more active approaches include working closely with government to help them transform their discourses using risk science. The article has wider implications in guiding policymakers on how they can elicit more value from risk science, not least by: identifying credible risk scientists; raising scientists’ awareness of the context of their models to continually enhance their relevance; directing scientific inquiry to address policy‐related issues; and being open to using the findings from risk models to create their evidence‐based arguments. Policymakers can also consider knowledge from local risk managers when developing and disseminating policy.

Foucault's early death prevented his illustration of subjectification with empirical examples (Alvesson & Skoldberg, 2017; Fairclough, 2005). So, to progress his early work into risk science to understand risk discourse and objectified‐subjectification is novel. Our findings complement Foucault's theories and illustrate how risk science evidence was modified through the art of Governmentality to gain the trust and obedience of citizens in COVID‐19. We also illustrate the foundational issue from this article—how the process of objectified‐subjectification to risk science discourse engenders a human response of resistance in policymakers, risk scientists, and risk managers. Risk management shows how the analysis of a disturbed system can identify new risks and uncertainties which can further disturb risk managers (as users of risk science) and risk scientists (as disturbed senders of new insights). Taking action to lower those new risks can reflect a subjectification process of social interaction where human agency resists the discourse. That agency, whilst struggling with uncertainty, considers the context to ensure that the best available action is taken (Althaus, 2005).

We identify as future work the opportunity to theorize the relevance of Foucault's concept of Governmentality and theory of discourse toward a deeper understanding of the contribution from risk science (Althaus, 2005). For example, while the findings in our article are informed by UK data, further work can use the concepts we present to explore the influence of risk science in other countries during COVID‐19 and during other crises. There are opportunities to explore how risk science can develop and amplify its discourses to benefit risk management, and how discourses in other domains may substantiate those (Aven & Bouder, 2020).

## APPENDIX A SOURCES OF SECONDARY DATA

### A.1

S1. BBC documentary (2020). Lockdown 1.0—Following the Science? *BBC Television* 19 November 2020. https://www.bbc.co.uk/programmes/m000pjr1

S2. Mangan, L. (2020). Lockdown 1.0: Following the Science? Review—Wikipedia, Wuhan and worrying mistakes. *The Guardian*. 19 November. https://www.theguardian.com/tv‐and‐radio/2020/nov/19/lockdown‐10‐following‐the‐science‐review‐wikipedia‐wuhan‐and‐worrying‐mistakes

S3. SAGE (2020a). SAGE Precautionary SAGE 1 min: Coronavirus (COVID‐19) response, 22 January 2020. UK Government. 22 January. https://www.gov.uk/government/publications/precautionary‐sage‐minutes‐coronavirus‐covid‐19‐response‐22‐january‐2020/precautionary‐sage‐1‐minutes‐coronavirus‐covid‐19‐response‐22‐january‐2020

S4. SAGE (2020b). SAGE 3 minutes: Coronavirus (COVID‐19) response. *Scientific Advisory Group for Emergencies, UK Government*. 3 February. https://www.gov.uk/government/publications/sage‐minutes‐coronavirus‐covid‐19‐response‐3‐february‐2020

S5. SAGE (2020c). SAGE 12 minutes: Coronavirus (COVID‐19) response. *Scientific Advisory Group for Emergencies, UK Government*. 3 March. https://www.gov.uk/government/publications/sage‐minutes‐coronavirus‐covid‐19‐response‐3‐march‐2020

S6. SAGE (2020d). SAGE 74 minutes: Coronavirus (COVID‐19) response. *Scientific Advisory Group for Emergencies, UK Government*. 22 December. https://www.gov.uk/government/publications/sage‐74‐minutes‐coronavirus‐covid‐19‐response‐22‐december‐2020

S7. Busby, M. (2020). Bar Dominic Cummings from Sage meetings, Labour urges. *The Guardian*. 25 April. https://www.theguardian.com/world/2020/apr/25/bar‐dominic‐cummings‐from‐sage‐coronavirus‐meetings‐labour‐urges

S8. Blakely, R. (2020). Sage panel shown to lack critical areas of expertise. *The Sunday Times*. 5 May.

S9. Whitty, C. (2020). Evidence provided to the Health and Social Care Committee Oral evidence: Management of the Coronavirus Outbreak, HC 36. *UK Government House of Commons*. 21 July.

S10. DHSC (2020). Coronavirus action plan launched. *Department of Health and Social Care, UK Government*. 3 March. https://www.gov.uk/government/news/coronavirus‐action‐plan‐launched

S11. Sample, I., and Stewart, H. (2021). “A class act”: Chris Whitty, the calm authority amid the Covid crisis. *The Guardian*, 22 March. https://www.theguardian.com/world/2021/mar/22/a‐class‐act‐sir‐chris‐whitty‐the‐calm‐authority‐amid‐covid‐crisis‐chief‐medical‐officer

S12. Blewett, S. (2022). Sir Patrick Vallance to stand down as chief scientific adviser. The Independent. 3 August. https://www.independent.co.uk/news/health/patrick‐vallance‐stand‐down‐chief‐scientific‐adviser‐b2137734.html

S13. Newton, J. (2020). Behind the headlines: Counting COVID‐19 deaths. *UK Health Security Agency, UK Government*. https://ukhsa.blog.gov.uk/2020/08/12/behind‐the‐headlines‐counting‐covid‐19‐deaths/

S14. Cabinet Office (2013). Emergency Response and Recovery. *HM Government*.

S15. DEFRA (2021). The role of convergent volunteers during a flood emergency. *Department for Environment, Food & Rural Affairs, UK Government*. 23 February. https://www.gov.uk/flood‐and‐coastal‐erosion‐risk‐management‐research‐reports/the‐role‐of‐convergent‐volunteers‐during‐a‐flood‐emergency

S16. DEFRA (2015). Spontaneous volunteers: Involving citizens in the response and recovery to flood emergencies. *Department for Environment, Food & Rural Affairs, UK Government*. July 2015.

S17. Independent SAGE website. www.independentsage.org

S18. JESIP (2022). Multi‐Agency Information Cell (MAIC) Guidance. *Joint Emergency Service Interoperability Programme* (JESIP), UK. 29 June.

S19. Bloom, D. (2021). Tory minister bizarrely declares “schools are safe but are vectors for coronavirus”. *The Mirror*. 6 January. https://www.mirror.co.uk/news/politics/tory‐minister‐bizarrely‐declares‐schools‐23269695

S20. The Andrew Marr Show (2021). Interview with Boris Johnson. *BBC Television*. 3 January.

S21. Weaver, M. (2021). UK health agency chief urges people to keep getting COVID boosters. *The Guardian*. 24 December. https://www.theguardian.com/world/2021/dec/24/uk‐health‐agency‐chief‐urges‐people‐keep‐getting‐covid‐boosters

S22. Vallance, P. (2020a). It's a national effort to win coronavirus fight, we all have crucial part to play. *The Sun Newspaper*. 16 March. https://www.gov.uk/government/speeches/its‐a‐national‐effort‐to‐win‐coronavirus‐fight‐we‐all‐have‐crucial‐part‐to‐play

S23. Vallance, P. (2020b). Written Evidence Submitted by Sir Patrick Vallance, UK Government Chief Scientific Adviser and Head of the Government, Science and Engineering Profession. *UK Government House of Commons*. 24 July. https://committees.parliament.uk/writtenevidence/10928/pdf/

S24. The Life Scientific (2021). The Patrick Vallance interview. *BBC Radio*. 12 October. https://www.bbc.co.uk/sounds/play/p09ydn63

S25. Mallinson, J. (2021). Mastery, the scientific method, and policy design. *UK Government*. 18 November. https://publicpolicydesign.blog.gov.uk/2021/11/18/mastery‐the‐scientific‐method‐and‐policy‐design/

S26. Newsnight (2021). Interview with Professor Susan Michie. *BBC Television*. 8 Jan 2021.

S27. Sky News (2020). Shutdown: The virus that changed the world. *SkyNews*. 5 June. https://www.youtube.com/watch?v=W3sisz6FA6A

S28. UK Government (2020a). Prime Minister's statement on coronavirus (COVID‐19): 22 March 2020. UK Government. 22 March. https://www.gov.uk/government/speeches/pm‐statement‐on‐coronavirus‐22‐march‐2020

S29. UK Government (2020b). Prime Minister's statement on coronavirus (COVID‐19): 23 March 2020. *UK Government*. 23 March. https://www.gov.uk/government/speeches/pm‐address‐to‐the‐nation‐on‐coronavirus‐23‐march‐2020

S30. UK Government (2020c). Prime Minister's statement on coronavirus: 25 March 2020. *UK Government*. 25 March. https://www.gov.uk/government/speeches/pm‐statement‐on‐coronavirus‐25‐march‐2020

S31. UK Government (2020d). Communities Secretary's statement on coronavirus (COVID‐19): 29 March 2020. *UK Government*. 29 March. https://www.gov.uk/government/speeches/communities‐secretary‐robert‐jenrick‐on‐covid19‐response

S32. UK Government (2020e). Foreign Secretary's statement on coronavirus (COVID‐19): 7 April 2020. UK Government. 7 April. https://www.gov.uk/government/speeches/foreign‐secretarys‐statement‐on‐coronavirus‐covid‐19‐7‐april‐2020

S33. UK Government (2020f). Home Secretary's statement on coronavirus (COVID‐19): 22 May 2020. UK Government. 22 May. https://www.gov.uk/government/speeches/home‐secretarys‐opening‐statement‐to‐the‐governments‐daily‐briefing‐on‐coronavirus

S34. UK Government (2020g). Prime Minister's statement on coronavirus (COVID‐19): 31 October 2020. UK Government. 31 October. https://www.gov.uk/government/speeches/prime‐ministers‐statement‐on‐coronavirus‐covid‐19‐31‐october‐2020

S35. UK Government (2021a). Prime Minister's address to the nation: 4 January 2021. *UK Government*. 4 January. https://www.gov.uk/government/speeches/prime‐ministers‐address‐to‐the‐nation‐4‐january‐2021

S36. UK Government (2021b). Prime Minister's statement on coronavirus (COVID‐19): 5 January 2021. *UK Government*. 5 January. https://www.gov.uk/government/speeches/prime‐ministers‐statement‐on‐coronavirus‐covid‐19‐5‐january‐2021

S37. UK Government (2021c). 138 transcripts of ministerial statements on coronavirus (COVID‐19). *UK Government*. 3 March 2020 to 23 June 2021. https://www.gov.uk/government/collections/slides‐and‐datasets‐to‐accompany‐coronavirus‐press‐conferences

S38. UK Government (2022a). 144 sets of slides, datasets and transcripts to accompany coronavirus press conferences. *UK Government*. 30 March 2020 to 21 February 2022. https://www.gov.uk/government/collections/slides‐and‐datasets‐to‐accompany‐coronavirus‐press‐conferences

S39. UK Government (2022b). 25 collections of meeting minutes and supporting papers from the Scientific Advisory Group for Emergencies (SAGE). *UK Government*. January 2020–February 2022. https://www.gov.uk/government/collections/scientific‐evidence‐supporting‐the‐government‐response‐to‐coronavirus‐covid‐19

S40. Demianyk, G. (2023). Boris Johnson Mocked for failing to grasp basic maths during pandemic*. The Huffington Post*. 1 March. https://www.huffingtonpost.co.uk/entry/boris‐johnson‐mocked‐for‐failing‐to‐grasp‐basic‐maths‐during‐pandemic_uk_63fe4fdae4b0cab1fa31bb0c

S41. Yates, K. (2023). Did Boris Johnson “follow the science” on Covid? He couldn't even do the maths. *The Guardian*. 3 March. https://www.theguardian.com/commentisfree/2023/mar/03/boris‐johnson‐science‐covid‐maths‐whatapps‐advisers

S42. Channel 4 (2020). The country that beat the virus: What can Britain learn? 13 May. https://www.channel4.com/programmes/the‐country‐that‐beat‐the‐virus/on‐demand/71452‐001

S43. Mangan, L. (2020). The country that beat the virus review—breathtaking, damning and humiliating. *The Guardian*. 13 May. https://www.theguardian.com/tv‐and‐radio/2020/may/13/the‐country‐that‐beat‐the‐virus‐review‐breathtaking‐damning‐and‐humiliating

S44. Independent SAGE website: https://www.independentsage.org/

S45. Your Questions Answered (2020). Interview with Professor Jenny Harries, UK Deputy Chief Medical Officer. *BBC News*. 12 March. https://www.facebook.com/bbcnews/videos/1295066484035973

S46. Thornton, (2021b). Health Protection (Coronavirus, Wearing of Face Coverings) (England) Regulations 2021. Volume 816. *UK House of Lords*. 1 December. https://hansard.parliament.uk/lords/2021‐12‐01/debates/4590DFA3‐95EC‐4E75‐935B‐71A5EA28C13B/HealthProtection(CoronavirusWearingOfFaceCoverings)(England)Regulations2021

S47. BBC Radio 4 Today (2020). Interview with Patrick Vallance. *BBC Radio 4*. 13 March. https://www.youtube.com/watch?v=bEoDWqSH488

S48. Gould, M., Joshi, I., Tang, M. (2020). The power of data in a pandemic. *Department of Health and Social Care, UK Government*. 28 March.

S49. C19 (2020a). COVID‐19 pandemic first interim operational review. *C19 National Group*. May.

S50. C19 (2020b). COVID‐19 pandemic second interim operational review. *C19 National Group*. July.

S51. C19 (2020c). COVID‐19 pandemic third interim operational review. *C19 National Group*. October.

## APPENDIX B SEMISTRUCTURED INTERVIEW PROTOCOL

### B.1

Using our Phase 1 insights, we carefully selected 22 interviewees to understand their objectified‐subjectification. To ensure consistency in our interviews, we focused each interviewee on a substantial project that they lead on behalf of the local government resilience partnership. Each project was initiated to satisfy a national government instruction to support communities during the pandemic.

To allow interviewees to explore their different perspectives, we took a semistructured approach to each interview. This allowed us to probe interviewees with follow‐up questions in different ways and in relation to the content of their answers.

The analysis was conducted to discover how the interviewees were objectified, how their human agency enabled their subjectification, and how their project enabled their process of objectified‐subjectification.

To understand objectification we explored their:

1. Motivations to initiate the project, and where those originated
2. Aims and objectives of the project, and where those originated
3. Rules and instructions that the project should follow, and where those originated

4. To understand subjectification we explored their:

i. Design and innovation of project actions
ii. From where permission to conduct actions was secured
iii. Different discourses that informed the project design and delivery
iv. Project leadership and intrapreneurship
v. Challenges to delivery, and how those were overcome

The semi‐structured interviews permitted interviewees to work through these issues and allowed interviewees to differently explore details around their involvement in risk management.

## APPENDIX C INTERVIEWEES FROM A UK LOCAL GOVERNMENT RESPONSE PARTNERSHIP THAT OPERATED TO MINIMISE COVID‐19 RISKS

### C.1

**Table:**

**Participant#**	**Risk management role in local government response partnership (LGRP)**
P1	Risk and resilience manager for the LGRP
P2	Voluntary sector executive informally seconded to LGRP to manage local risks and responses
P3	Voluntary sector executive informally seconded to LGRP to manage local risks and responses
P4	Risk and resilience manager for the LGRP
P5	Risk and resilience manager for the LGRP
P6	Risk and resilience manager for the LGRP
P7	Risk and resilience manager for the LGRP
P8	Chair of COVID‐19 community group working closely with LGRP to manage local risks and responses
P9	Risk and resilience manager for the LGRP
P10	Risk and resilience manager for the LGRP
P11	Risk and resilience manager for the LGRP
P12	Business executive informally seconded to LGRP to manage local risks and responses
P13	Business manager informally seconded to LGRP to manage local risks and responses
P14	Business manager informally seconded to LGRP to manage local risks and responses
P15	Business manager informally seconded to LGRP to manage local risks and responses
P16	Voluntary sector manager informally seconded to LGRP to manage local risks and responses
P17	Voluntary sector executive informally seconded to LGRP to manage local risks and responses
P18	Voluntary sector manager informally seconded to LGRP to manage local risks and responses
P19	Voluntary sector executive informally seconded to LGRP to manage local risks and responses
P20	Voluntary sector manager informally seconded to LGRP to manage local risks and responses
P21	Voluntary sector executive informally seconded to LGRP to manage local risks and responses
P22	Chair of COVID‐19 community group working closely with LGRP to manage local risks and responses
